# Diffusion‐Weighted MRI Brain Connectivity Assessment of Healthy Aging Trajectories in Marmosets

**DOI:** 10.1002/alz.093921

**Published:** 2025-01-09

**Authors:** Diego Szczupak, Rebecca Bhik‐Ghanie, Bei Zhang, Daniel Papoti, Vinicius Campos, Lauren R Dubberley, T Kevin Hitchens, Fang‐Cheng Yeh, Lauren K Hayrynen Schaeffer, Gregory W Carter, Stacey J Sukoff Rizzo, David J Schaeffer, Afonso C Silva

**Affiliations:** ^1^ University of Pittsburgh School of Medicine, Pittsburgh, PA USA; ^2^ Universidade de Sao Paulo, Sao Carlos, Sao Paulo Brazil; ^3^ The Jackson Laboratory, Bar Harbor, ME USA

## Abstract

**Background:**

The common marmoset (Callithrix jacchus) is an important animal model in neuroscience and neurological diseases (e.g., Alzheimer’s disease ‐ AD), as they present primate‐specific evolutionary features such as an expanded frontal cortex. We established a new consortium with funding support from the National Institute on Aging to generate, characterize, and validate MArmosets as Research MOdels of AD (MARMO‐AD). This consortium develops and studies gene‐edited marmoset models carrying genetic risk for AD, comparing them against wild‐type aging marmosets from birth throughout their lifespan, using non‐invasive longitudinal assessments. Here, we aimed to characterize the structural cortical connectivity (white matter fibers) in a population of marmosets across the lifespan to establish healthy aging trajectories against which we will compare our genetically engineered marmoset models of AD.

**Method:**

We performed high‐resolution (500 µm isotropic) diffusion‐weighted MRI (dMRI) in a cohort of 19 marmosets (13 males, 6 females) aged 8 to 89 months using a dedicated 9.4T 30cm bore MRI scanner (Bruker BioSpin Corp, Billerica). The animals were anesthetized under isoflurane and maintained at normal physiological conditions. The brain images were aligned and registered to the Marmoset Brain Mapping (MBM) V2 template. The brain was segmented using the MBM white matter atlas, and voxel‐based morphology (VBM) was used to quantify regional white matter volume in the left and right hemispheres. Furthermore, we used DSI STUDIO to perform the whole‐brain tractogram, calculate network‐based statistics (NBS), and correlate them with age.

**Result:**

We measured decreases in the NBS properties of Assortativity, Small Worldliness, and Hierarchy with age but an increase in Efficiency, showing the maturation processes of white matter across development. We are actively working on increasing our sample size to identify the entire marmoset age trajectory and its impact on white matter connectivity.

**Conclusion:**

Our work is the first to thoroughly describe the healthy aging trajectories of the marmoset brain, a valuable model for age‐related neuropathologies (e.g., AD). This research will establish normative baselines for changes in marmoset brain connectivity with aging that will be used to evaluate our genetically engineered marmoset models of AD, which is the goal of the MARMO‐AD consortium.

